# What is known about changes in pelvic floor muscle strength and tone in women during the childbirth pathway? A scoping review

**DOI:** 10.18332/ejm/189955

**Published:** 2024-08-02

**Authors:** Andrea Manzotti, Simona Fumagalli, Sonia Zanini, Veronica Brembilla, Adele Alberti, Ilaria Magli, Elis Buratti, Nicoletta Coraglia, Andrea De Fusco, Daniel Zambù, Valeria Zanotta, Antonella Nespoli

**Affiliations:** 1 RAISE Lab, Clinical-Based Human Research Department, Foundation COME Collaboration, Pescara, Italy; 2 Research Department, SOMA, Istituto Osteopatia Milano, Milan, Italy; 3 University of Milano-Bicocca, Monza, Italy

**Keywords:** pelvic floor, pregnancy, childbirth, distensibility, strength, midwifery care

## Abstract

**INTRODUCTION:**

This scoping review aims to comprehensively explore the existing research on the changes in pelvic floor function that occur throughout the childbirth pathway (antenatal, intrapartum, and postnatal period). Furthermore, it seeks to identify new opportunities and directions for future research in this field. In particular, this review focuses on investigating pelvic floor muscle strength and tone in women during the childbirth pathway.

**METHODS:**

The following databases were investigated from their inception: PubMed, OVID, Medline, ScienceDirect, The Cochrane Central Library, Scopus, Web of Science, PEDro, Scholar Google, Embase, and CINHAIL. Literature research was carried out from March to October 2022. Records identified through database searching were imported to Covidence. According to Arksey and O’Malley’s five-stage scoping review framework, researchers screen titles and abstracts for eligibility and exclude records that do not meet the inclusion criteria.

**RESULTS:**

A total of 40 studies were included in the data extraction phase. These articles underwent a review, with a specific emphasis on examining the tone, strength, and distensibility of the pelvic floor throughout the childbirth pathway. Among the selected studies, 22 investigated pelvic floor strength, 7 the distensibility, and 3 articles the tone.

**CONCLUSIONS:**

This review identified both areas of agreement and disagreement across all three themes examined, with a particular emphasis on labor and the postpartum period. Notably, the review unveiled a significant scarcity of data concerning the tone of pelvic floor muscles throughout the childbirth pathway. Further studies to investigate the relationship between midwifery care and pelvic floor outcomes are required to improve clinical practice.

## INTRODUCTION

Pelvic floor support is a highly dynamic structure that is kept in balance by pelvic floor muscles, endopelvic fascia, connective tissue, and pelvic bones. Any changes in these factors may cause an unpredictable imbalance in the pelvic floor, which emerges as pelvic floor disorders (PFDs), including pelvic organ prolapse (POP), incontinence, and sexual dysfunction^1^. PFDs are associated with a negative impact on quality of life and healthcare spending, and their impact is expected to grow as the prevalence of these disorders increases with the aging of the population^2^.

The weakness of the pelvic floor muscles (PFMs) is a common cause of pelvic floor dysfunction after childbirth^3^. Pregnancy, vaginal birth, parity, duration of the second stage of labor, difficulty extracting the fetus during a cesarean section (CS), infant weight, perineal trauma, and other mechanical, endocrine, and neural factors can lead to reduction or loss of pelvic floor muscle tone causing genitourinary disorders^4-7^. Regarding birth, it is likely that the risk of pelvic floor trauma may be influenced by the intrinsic characteristics of the pelvic floor muscles and their ability to stretch sufficiently to allow the passage of the fetus through the birth canal without damage^8^. Furthermore, it has been reported that partial denervation in the pelvic floor may occur, especially in the first pregnancy, and the risk of PFD increases with the severity of the damage in most women with vaginal birth^9^. Nevertheless, it is still a matter of debate as to what extent pelvic floor changes are caused by vaginal birth and birth trauma and to what extent they can be attributed to pregnancy-specific changes^10^.

To date, there is an overall paucity in the literature regarding the nature and timing of pelvic floor changes in the human pregnant and postpartum states, due to a lack of prospective cohort studies of subjects through pregnancy and into the postpartum state^11^. This scoping review intends to comprehend the current research landscape about changes in pelvic floor function during the childbirth pathway (antenatal, intrapartum, and postnatal period) and identify new frontiers for future research in this field.

## METHODS

### Research aims

This scoping review sought to explore the literature related to changes in pelvic floor muscle during the childbirth pathway (antenatal, intrapartum, and postnatal period). Specifically, the review intended to show the muscle strength and tone in women during the childbirth pathway.

### Research strategies

The study was conducted in collaboration with physiotherapists, osteopaths, midwives, and researchers from Foundation COME, SOMA, and School of Medicine and Surgery University of Milano Bicocca. The research protocol was registered on the Open Science Framework (OSF) platform on 6 July 2022. As a ‘rigorous and transparent method for mapping areas of research’ but which permits ‘subjective interpretation’ of existing knowledge^12^, the review broadly followed the five-stage scoping review framework of Arksey and O’Malley^13^.

The PRISMA ScR Extension Fillable Checklist^14^ was used to promote robust data collection, analysis, and reporting.

After defining the research question (phase 1), the authors identified relevant studies and developed a decision plan for the search strategy (phase 2). The following databases were searched from their inception: PubMed, OVID, Medline, ScienceDirect, The Cochrane Central Library, Scopus, Web of Science, PEDro, Scholar Google, Embase, and CINHAIL. Literature research was carried out from March to October 2022.

Text mining was undertaken to identify keywords, synonyms, and MeSH terms, which were identified and included in the search strategy. Keywords were combined using AND and OR Boolean operators in search strings, and truncation was used when required. Search strings (Supplementary file Table 1) were developed around the following key concepts:

1) childbirth pathway, which includes the prenatal period: pregnancy, labor, mode of birth, and postnatal period (6 weeks); and 2) field of evaluation of pelvic dysfunction and muscular stress: pelvic, perineum dysfunction, muscular disorder, stress, muscle strength, muscle tone.

During the study selection phase 3, authors identified post hoc inclusion and exclusion criteria. Articles were included in the review if they met the following criteria: systematic reviews, randomized and not randomized experimental trials, prospective and retrospective cohort studies, review studies, case-control studies, observational and case report studies; studies that consider the assessment of the female pelvic floor during the period of time that includes pregnancy, birth, and puerperium; studies that include pelvic floor evaluation as the primary intervention for participants; and studies written in English or Italian. Exclusion criteria were studies relating to women with high-risk pregnancies, letters to the editor, and grey literature.

All the articles identified during the search were imported into Covidence for review. Covidence is a web-based platform used to facilitate screening and data extraction for systematic reviews (Covidence, 2019).

Two researchers (VB and SZ) independently screened the titles and abstracts of all retrieved citations for inclusion in the full-text review. Any doubts about inclusion were referred to the arbiter (AA) for consensus. Subsequently, two reviewers (AM and SF) independently assessed the articles selected for full-text review to determine if they met the inclusion/exclusion criteria. The reference lists of the studies included in this review were manually screened for additional studies. During the full-text screening process, additional exclusion criteria were applied: 1) irrelevant focus, 2) inability to locate the full text, and 3) duplicates that have not been identified previously. Any discordant full-text articles were reviewed a second time, and further disagreements were resolved through discussion with a third investigator (AN) until a consensus was reached. The findings of the analysis were discussed with the broader research team at regular research meetings, and their insights were incorporated into the analysis until a complete consensus was reached.

In phase 4, articles meeting the inclusion criteria were reviewed, and data were extracted and charted pertaining to research design, study’s objective, population characteristics (parity, low-high risk), stage of childbirth pathway (antenatal, intrapartum and postnatal period), mode of birth, intrapartum variables (perineum status), inclusion and exclusion criteria, practitioner information, outcomes description, main results, limitations and side effects and adverse events reported. The primary elements from each source were extracted and then compared using a structured Data Extraction Form for data synthesis. This information was used to develop the narrative by identifying themes and mapping the literature. A narrative analysis was chosen over a descriptive analysis to understand better the significance of the findings beyond the scope of simply describing them (phase 5).

## RESULTS

**Figure 1:**
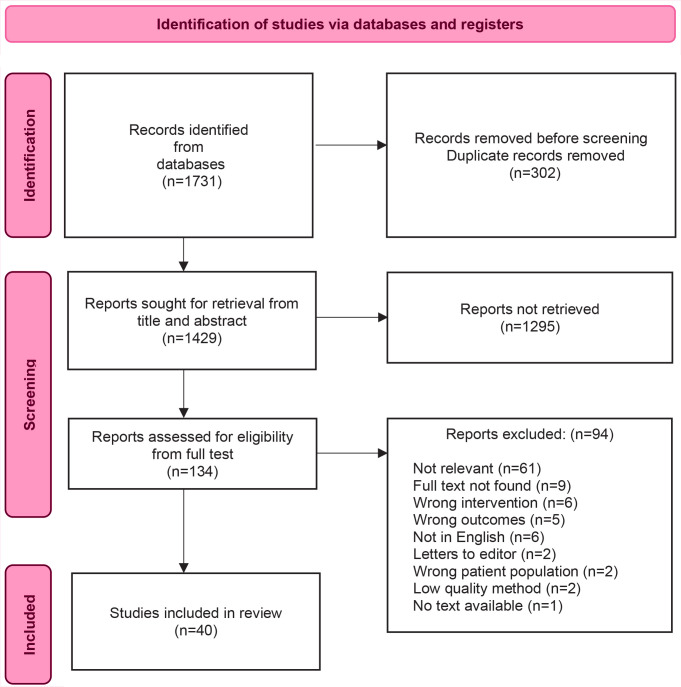
PRISMA flow chart

The searches retrieved 1731 articles. Then, 1429 records were screened following deduplication, resulting in 134 articles being screened. No additional studies were found in the review of reference lists, resulting in a total of 40 articles analyzed. Of these, 39 studies were quantitative, and one was a systematic review. The PRISMA flow diagram describes the screening and selection process (Figure 1).

Of the 40 selected articles, in addition to the primary outcome of pelvic floor muscle strength and tone modification during childbirth, another parameter was identified: distensibility. This parameter has been considered important for the evaluation of the pelvic floor because many articles studied it, so it was included in the scoping as an additional outcome for the modification of the PFM. The articles are, therefore, divided in the scoping review according to the parameter considered. Table 1 provides a description of the included articles, while in Table 2, a summary of the articles is presented based on the parameter treated in relation to the period of the birth path analyzed or to the presence of specific regulations.

**Table 1:** Characteristics of studies involved in the scoping review

*Study*	*Population*	*Intervention*	*Comparison*	*Outcomes*	*Results*
**Ali et al.**^38^ **2022** Study design: Cohort study Country: Pakistan AIM: To evaluate the influencing pelvic floor changes on the quality of women’s life during and post pregnancy	Sample size: 45 primigravidae women’s Inclusion criteria: All primigravida women with an uncomplicated full-term pregnancy.	Intervention: Pre-designed questionnaire Vaginal examination Translabial 2D/3D ultrasonography POP-Q test In the 3rd trimester	Comparison: Pre-designed questionnaire Vaginal examination Translabial 2D/3D ultrasonography POP-Q test Three months after birth	Outcomes: Ultrasound measures of pelvic floor anatomic alterations and clinical evaluation. Potential restrictions in quality of life.	Results: Weakness of connective tissues was observed in 23 (51.1%) Pelvic floor dysfunction is associated with connective tissue weak and a larger fetus weight.
**Botelho et al.**^15^ **2010** Study design: Prospective, longitudinal and comparative study Country: Brazil AIM: To evaluate pelvic floor muscle function of the pelvic floor during the last trimester of pregnancy and 45 days after delivery	Sample size: 75 primiparous women Inclusion criteria: Primiparous women randomly selected between Feb. 2006 and Feb. 2008.	Intervention: All patients underwent vaginal digital examination, grading the muscle contractility from 0 to 5 and surface electromyography (EMG) of the pelvic floor during the last trimester of pregnancy (mean 32 week)	Comparison: The same evaluation at 45 days after delivery (10 days), comparing vaginal, elective cesarean section and emergency cesarean section.	Outcomes: To verify, in a more accurate way, the effect of mode of delivery on pelvic floor muscles.	Results: There was a significant increase in pelvic floor muscle contractility after delivery in the elective cesarean section group (p= 0.03), and a significant loss of muscle contractility evaluated by electromyography in patients who underwent vaginal delivery (p=0.001).
**Brunelli et al.**^39^ **2020** Study design: Prospective cohort study Country: Italy AIM: To evaluate the correlation between the proportional change of anteroposterior diameter (APD) of levator hiatus from rest to maximum Valsalva maneuver in nulliparous and labor outcome.	Sample size: 495 women were recruited in the study. 486 women were included in the analysis. Inclusion criteria: Nulliparous women at term (37-41 weeks’ gestation) cephalic presentation.	Intervention: two-dimensional transperineal ultrasound, measuring the APD of the elevator hiatus at rest and under maximum Valsalva’s maneuver. Proportional change of anteroposterior diameter (APD)	Comparison: **_**	Outcomes: Mode of delivery. Correlation of APD change with the mode of delivery and with labor durations was assessed.	Results: Significant negative relationship between the change of the APD of the levator hiatus from rest to Valsalva in nulliparous women at term and the duration of the active second stage of labor: the higher the increase of APD with Valsalva the shorter was the active second stage of labor.
**Caroci et al.**^16^ **2010** Study design: Analytic-correlational study Country: Brazil AIM: compare the measurements of women’s PFMS during pregnancy and postpartum period.	Sample size: The final sample included 110 women who completed all four stages of the study. Inclusion criteria: Be nulliparous in the first 12 weeks of pregnancy.	Intervention: The pelvic floor musculature strength was evaluated by perineometry (PERITRON) and digital vaginal palpation were followed at 12 weeks of pregnancy.	Comparison: The same evaluations were followed between 36-40 weeks of pregnancy; within 48 hours after childbirth; 42-60 days after childbirth.	Outcomes: Pelvic floor muscle strength reduction during pregnancy and childbirth.	Results: The PFMS of the women did not change significantly during pregnancy or after delivery (p= 0.78). In all three examined stages, a low-intensity pelvic floor musculature strength was prevalent with scores from 0-3.
**Caroci et al.**^17^ **2014** Study design: Cross-sectional study Country: Brazil AIM: Analyze the PFMS of pregnant women who had one or more vaginal or cesarean deliveries according to age, type of delivery, number of previous vaginal deliveries, perineal conditions, BMI.	Sample size: 110 pregnant women with vaginal deliveries or CS and 110 primigravidae. Inclusion criteria: All women with one or more previous vaginal or cesarean deliveries at 12 weeks.	Intervention: Evaluation of PFMS (Pelvic Floor Muscle Strength) was evaluated by perineometry (Peritron) and vaginal digital palpation (modified Oxford Scale) in pregnant woman with one or more vaginal deliveries or cesarean sections.	Comparison: Pregnant women at their first pregnancy.	Outcomes: Compare PFMS of the two groups of study.	Results: The PFMS of the majority of pregnant women was classified as weak in the first trimester. The PFMS, when evaluated by vaginal digital palpation, presented a statistically significant lower degree among women who had undergone one or more previous deliveries, both vaginal and cesarean, compared with the primigravidae.
**Cetindag et al.**^1^ Study design: Analytic-correlational study Country: Turkey AIM: To document the deterioration in pelvic organ support occurring throughout all trimesters during the first pregnancy of women with no known risk factors.	Sample size: 41 primigravid women with a singleton pregnancy in first trimester (< 14 w) Inclusion criteria: Turkish-speaking primigravidae with singleton, > 18 years old.	Intervention: During follow-up pelvic organ support changes were documented by using POP-Q system. Pelvic floor muscle strength examination, by modified Oxford scoring (MOS), and symptom assessment by Pelvic Floor Distress Inventory-Short Form (PFDI-20) in first trimester.	Comparison: The same evaluation in second (T2), and third trimester (T3).	Outcomes: Differences in tests POPQ MOS PFDI-20	Results: No significant difference in Modified Oxford Scale was observed throughout pregnancy.
**Chen et al.**^40^ **2013** Study design: Prospective observational cohort study Country: China AIM: Compare changes in pelvic organ prolapse (POP) in women undergoing unlabored cesarean section (UCD) or trial of labor (TOL) from pregnancy to 1-year postpartum in nulliparous.	Sample size: A total of 108 nulliparous women who were at 36–38 weeks of gestation and were planning to undergo an elective cesarean delivery or a TOL. Inclusion criteria: All women with a normal, uncomplicated single gestation.	Interventions: Pelvic organ prolapse (POP) was assessed at 36-38 weeks of gestation, then at 6 weeks, 6 months, and 1 year postpartum, using the Pelvic Organ Prolapse Quantification (POPQ) system.	Comparison: UCD group: women who were scheduled for and underwent a cesarean delivery before the onset of labor. TOL group: women who underwent a trial of labor.	Outcomes: Postpartum POP status in UCD and TOL determined by POP Q measurements over time.	Results: Confirm that long-lasting changes occur in pelvic floor architecture during pregnancy that lead to a significantly increased risk of sustained pelvic floor relaxation after labor and delivery.
**Driusso et al.**^18^ **2020** Study design: Systematic review Country: Brazil AIM: Investigate whether there was a difference in short-term PFM function after childbirth in primiparous women who underwent cesarean section compared with those who underwent vaginal delivery.	Sample size: 11 studies A total of 1726 primiparous women were analyzed after childbirth. Five studies were included in the meta-analysis.	Interventions: PFM function in primiparous women who underwent cesarean section.	Comparisons: PFM function in primiparous women who underwent vaginal delivery.	Outcomes: Short-term PFM function after childbirth	Results: No differences in short-term PFM strength after birth were identified among primiparous women who underwent cesarean section or vaginal delivery. However, PFM strength was lower for women who underwent episiotomy or instrumented vaginal delivery compared with those who underwent cesarean section.
**Dua et al.**^41^ **2009** Study design: Prospective, observational study Country: UK AIM: To generate normative data for perineal length for Caucasian and Asian women in labor.	Sample size: 1000 women in the first stage of labor Inclusion criteria: All women in labor were eligible to participate in the study. Exclusion criteria: Women who underwent elective or emergency cesarean section.	Interventions: The distance from the posterior fourchette to the center of the anal orifice was measured. All measurements were performed using a standard disposable tape measure in Caucasian woman.	Comparisons: Asian women	Outcomes: Perineal integrity and perineal length	Results: Significantly higher incidence of thirddegree perineal tears in women with short perineum. Normal perineal length for Caucasian (3.7 cm) and Asian (3.6 cm) women in labor.
**Elenskaya et al.**^19^ **2011** Study design: Sub-analysis of a longitudinal observational cohort study Country: UK AIM: to evaluate PFMF during the second and third trimesters of pregnancy and after childbirth using subjective and objective methods.	Sample size: Four hundred and three women (182 nulliparous and 221 multiparous) attended visit. Inclusion criteria: English-speaking women over 18 years of age	Interventions: Digital palpation of the PFM using the modified Oxford scale and perineometry were performed during the second trimester (visit 1)	Comparisons: Evaluation in third trimester (visit 2), 14 weeks postpartum (visit 3) and 12 months postpartum (visit 4).	Outcomes: Variation of Oxford scale and perineometry assessments of PFMF at 20 and 36 weeks’ gestation and at 14 weeks and 12 months after delivery.	Results: Two hundred ninety-four (73%) delivered vaginally and 92 (23%) by caesarean section. RP and MSP improved significantly (p<0.01) during pregnancy. After childbirth, a significant decrease in PFMF was demonstrated, which recovered completely by 1 year in majority of women.
**Falkert et al.**^42^ **2013** Study design: Observational descriptive study Country: Germany AIM: To evaluate the relationship between persisting pelvic floor disorders 18–24 months after the first delivery, 3D ultrasound biometry and mode of delivery.	Sample size: 130 Caucasian primipara Inclusion criteria: Caucasian primipara with singleton pregnancy with cephalic presentation were recruited during their hospital stay on the second day after delivery.	Interventions: 3D transperineal ultrasound on the second day after delivery. Obstetric and constitutional parameters were obtained from our clinical files.	Comparisons: 3D translabial US 18-24 months after delivery and a standardized questionnaire was used to evaluate persisting pelvic floor disorders.	Outcomes: The relationship between persisting pelvic floor disorders (i.e. incontinence, pelvic organ prolapse, dyspareunia) 18–24 months after the first delivery.	Results: This study demonstrates that persisting morphological changes of the female pelvic floor can be imaged by 3D ultrasound techniques 2 years after first delivery. Larger hiatal dimensions were significantly correlated to vaginal delivery and persisting stress urinary incontinence.
**Frederice et al.**^20^ **2013** Study design: Cross-sectional study Country: Brazil AIM: To evaluate pelvic floor muscle (PFM) function and its association with urinary symptoms in the third trimester of pregnancy.	Sample size: 91 nulliparous women at 30–34 weeks of pregnancy Inclusion criteria: Pregnant women aged 18–35 years, between 30 and 34 weeks of pregnancy, with one fetus, were invited to participate.	Interventions: PFM was evaluated by surface electromyography (sEMG) and manual muscle testing, while urinary symptoms were identified by interview in third trimester of pregnancy.	Comparisons: _	Outcomes: Basal tone muscle strength and the correlation with urinary continence.	Results: Average sEMG values were 4.8 mV for basic tonus (BT), 19.2 mV for maximum voluntary contraction (MVC), and 12.9 mV for average sustained contraction (ASC), and 48.4% presented muscle strength grade 3. Nocturia was reported by 80.2%,
**Gachon et al.**^52^ **2017** Study design: Prospective longitudinal study Country: France AIM: To investigate a possible association between peripheral ligamentous laxity and levator hiatus (LH) distension during pregnancy.	Sample size: 30 women were included before the 12th w. 11 were older than 30 years, 7 were overweight and 19 were multiparous. Inclusion criteria: Pregnant, >18 years, and were followed in physiological antenatal clinics, regardless of their parity.	Interventions: 8–12 weeks, occurrence of pelvic organ prolapse (POP) symptoms, 4D perineal ultrasound scan results with LH distension assessment and measurement of metacarpophalangeal joint mobility (MCP laxity).	Comparisons: 20–24 weeks, and at 30–34 weeks of pregnancy	Outcomes: Peripheral ligamentous laxity and levator hiatus (LH) distension during pregnancy and their association.	Results: MCP laxity increased with pregnancy trimester and was associated with LH distension and POP symptoms suggesting, as expected, an association between peripheral ligamentous laxity and pelvic organ mobility during pregnancy.
**Gray et al.**^21^ **2020** Study design: Observational study Country: USA AIM: To investigate the relationship among muscle activities of the UT, AB, and PFMs by simultaneously recording their EA during the final steps of human parturition.	Sample size: 28 women with term pregnancies in labor	Interventions: Using EMG to evaluate muscular activities	Comparisons: **_**	Outcomes: **_**	Results: Childbirth muscular processes result from the coordinated UT and AB forces that must overcome the resistance of PFMs. Fetal head distension of PFMs contributes to stretch-induced EA and a resultant increase in the PFM resistance to delivery of the fetus. Contractility of PFMs contributes to normal labor processes.
**Guntinas et al.**^43^ **2022** Study design: Case control study Country: Spain AIM: To evaluate the characteristics of the pelvic floor, assessed by transperineal ultrasound, which can influence or increase the possibility of having a cesarean delivery.	Sample size: 97 patients, 80 deliveries were performed vaginally and 17 CS. Inclusion criteria: Nulliparous patients, aged 18–45 years, gestation between 10 and 16 weeks, we have included CS performed because of the failure of induction, non-progression of labor or cephalic pelvic disproportion.	Interventions: CS (Cesarean Section)	Comparison: VD (Vaginal delivery)	Outcomes: We define the elongation capacity (distensibility) of the levator ani muscle as the ratio between the area of the hiatus on Valsalva and the area of the hiatus at rest. In turn, we defined its contractility as the ratio between the area of the hiatus on PFMC and the area.	Results: In early pregnancy, the range of the resting hiatal area was 13.8 cm2 for cesarean sections, compared to 16.2 for vaginal deliveries with an OR of 0.57 For hiatal area on Valsalva, the OR was 0.55. Therefore, the smaller the hiatal area, the greater the possibility of cesarean section. At the end of pregnancy, 34-36 weeks of gestation, the OR of hiatal area on Valsalva was 0.78.
**Halle et al.**^22^ **2020** Study design: Analytic-correlational study Country: Norway AIM: Investigate the change in prevalence of major LAM defects from 6 weeks to 1 year postpartum, and second to assess maternal and obstetric risk factors for having persistent major LAM defects at 1 year pp.	Sample size: The study sample consisted of 212 primiparous women. Inclusion criteria: Nulliparity, singleton pregnancy, at 17-19 weeks and the ability to speak and understand a Scandinavian language.	Interventions: 21 weeks of gestation transperineal ultrasonography. Vaginal resting pressure, PFM and endurance.	Comparisons: 6 weeks 1 year postpartum	Outcomes: Change in prevalence of major LAM defects	Results: There was a 50% reduction of sonographically diagnosed major LAM defects from 6 weeks to 1 year postpartum. Long second stages of labor, high neonatal birthweight and vacuum delivery were associated with persistent major LAM defects/ avulsions.
**Hilde et al.**^23^ **2013** Study design: Analytic-correlational study. Country: Norway AIM: Study impact of childbirth and mode of delivery on PFM function in terms of ability to contract, VRP, and PFM strength and endurance from mid-pregnancy to 6 weeks postpartum.	Sample size: 277 nulliparous women Inclusion criteria: Nulliparous pregnant women Nulliparous women with a singleton pregnancy.	Interventions: Manometer was used for PFM measurements; differences were analyzed by t test (within groups) and analysis of variance in mid pregnancy.	Comparisons: Mode of delivery: NVD normal vaginal delivery IVD instrumental vaginal delivery Timing: 6 weeks postpartum	Outcomes: Ability to contract VRP PFM strength (as the mean of 3 maximal voluntary contraction) PFM endurance	Results: Pronounced reductions in VRP and in PFM strength and endurance were found after vaginal delivery. Continent women were stronger than incontinent counterparts.
**Jean Dit Gautieret al.**^36^ **2018** Study design: Case study Country: France AIM: Investigation of the use of an MRI-generated numerical model of the pregnant woman at different gestational ages and postpartum to analyze geometrical changes.	Sample size: A volunteer patient underwent MRI at 16, 32, and 38 weeks of gestation (wg) and 2 months and 1 year after delivery (postpartum). Inclusion/exclusion criteria: _	Interventions: We developed a parturient numerical model to assess pelvic structures at different gestational stages (16, 32, and 38 weeks) and postpartum (2 months and 1 year) using magnetic resonance imaging (MRI).	Comparisons: _	Outcomes: We studied changes in the length of uterosacral ligaments (USL) and thickness of the puborectal portion of the levator ani muscle (LAM) during and after pregnancy.	Results: This analysis brings some new elements and a new focus for discussion relating to changes in a parturient’s pelvic ligaments and muscles that are not simply linked to the increase in volume and size of the uterus. It could explain some clinical changes in status during and after pregnancy.
**Karahan et al.**^37^ **2018** Study design: Analytic-correlational study Country: Turkey AIM: To investigate the behavior of PFM during UC in spontaneous and induced labor in term pregnant women.	Sample size: 136 voluntarily term pregnant women Inclusion/exclusion criteria: Woman without any apparent obstetrical or other medical risk factors and expected to experience normal spontaneous labor, were included in the study	Interventions: The electrical activities of pelvic floor muscles (PFM) were recorded at rest and during contractions electromyographically in oxytocin infusion during labor.	Comparisons: The same evaluation in control group depending on clinical decision.	Outcomes: Behavior of PFM with EMG measurements.	Results: During physiological contractions the predominant behavior of PFM was a relatively silent status. More intense and more painful contractions caused by oxytocin seem to interfere with this coordination because of the physiological contractile reflexes of PFM in response to pain.
**Klein et al.**^24^ **1997** Study design: RCT Country: Canada AIM: To evaluate risk factors for severe vaginal-perineal trauma and to ascertain determinants of pelvic floor strength.	Sample size: 459 nulliparous women. Inclusion/exclusion criteria: Nulliparous women 17 to 40 years old, were para 0, 1, or 2, carried a single fetus, and were considered to be at no apparent risk by their physicians.	Interventions: Electromyographic perineometry was performed at enrolment and at 3 months postpartum as follows: women with sulcus tears	Comparisons: Women with concurrent third- or fourth-degree tears were excluded from the sulcus group to isolate factors that only have an impact on sulcus tears -women who did not have sulcus tears.	Outcomes: Examination of the association between sulcus tears, third- or fourthdegree tears, and pelvic floor strength and selected demographic, physiologic, pregnancy-related, and intrapartum factors.	Results: Determinants of sulcus tears appear to be present before pregnancy; third-and fourth-degree tears are related to physician management. Exercise mitigates the potential for severe trauma induced by episiotomy.
**Hock et al.**^25^ **2019** Study design: Observational descriptive study Country: Hungary AIM: It is known that incontinence rarely develops during pregnancy. The authors examined pelvic floor muscle function changes during pregnancy.	Sample size: 156 women Inclusion criteria: young nulliparous and pregnant women	Interventions: On examination of the muscle group the results of maximum muscle strength and duration of maximum muscle strength were processed. In order to measure the endurance, 75% of maximum perceived strength was calculated. Questionnaires were always filled.	Comparisons: First group involved young nulliparous women: number of examined women 52, the second group involved pregnant women in the 2nd and 3rd trimesters. The number of examined pregnant women is 104, average week of pregnancy: 28.6 weeks	Outcomes: Variation on muscle strength during pregnancy	Results: Significant differences were found concerning maximum voluntary contraction (p=0.002) and duration of maximum muscle contraction (p=0.012) in young nulliparous women compared to average results of pregnant women. This result can be proved in nulliparous women and among pregnant women in the 2nd (p=0.045), and 3rd trimesters (p=0.005).
**Meyer et al.**^26^ **2000** Study design: Analytic-correlational study Country: Switzerland AIM: To assess the effects of forcepsassisted delivery and spontaneous vaginal delivery on urethral sphincter and pelvic floor function in nulliparous women investigated during pregnancy and at 9 weeks and 10 months after delivery.	Sample size: 151 white nulliparous women Inclusion criteria: Nulliparous women that gave informed consent.	Intervention: Investigations with a questionnaire, clinical examination, assessment of bladder neck behavior, urethral sphincter function, intravaginal fissures during pelvic floor contractions.	Comparisons: Group 1 women with forceps deliveries, Group 2 of women with spontaneous vaginal deliveries	Outcomes: The incidence of stress urinary incontinence -the incidence of fecal incontinence -the decreased sexual response -bladder neck behavior, urethral sphincter function -intra-vaginal pressure -intra-anal pressure -pelvic floor weakness	Results: 10 months after delivery, the incidence of a weak pelvic floor (p=0.05) and the decrease in intraanal pressure between the pre- and post-delivery values (p=0.04) were significantly greater in the forceps-delivered women.
**Meyer et al.**^27^ **2017** Study design: Biomechanical clinical study Country: Switzerland AIM: To define a critical pressure threshold for the occurrence of permanent PF injury.	Sample size: 43 women with spontaneous delivery (group one) and in 17 women with forceps-assisted delivery (group two) Inclusion criteria: Everyone who gave informed consent during routine antenatal visits.	Interventions: In spontaneous delivery group: evaluation of the duration of bearing-down efforts.	Comparisons: Forceps-assisted delivery	Outcomes: The pressure parameters of PF and the ICS–validated questionnaires Urogenital Distress Inventory	Results: Postpartum PF complaints were not significantly different between the groups. The first and second phases of labor were longer in women of group two.
**Mørkved et al.**^28^ **2004** Study design: RCT Country: Norway AIM: To measure pelvic floor muscle function in continent and incontinent nulliparous pregnant women.	Sample size: 103 nulliparous pregnant women at 20 weeks of pregnancy Inclusion: Nulliparous, 18 years or more and had a singleton live fetus at a routine ultrasound scan at 18 weeks of pregnancy.	Interventions: Function was measured by vaginal squeeze pressure (muscle strength) and increment in thickness of the superficial pelvic floor muscles (urogenital diaphragm) assessed by perineal ultrasound.	Comparisons: Continent women Incontinent women	Outcomes: Measurement of thickness of the superficial pelvic floor muscle	Results: Continent women had statistically significantly higher maximal vaginal squeeze pressure and increment in muscle thickness when compared with incontinent women. Strong correlation between measurements of vaginal squeeze pressure and perineal ultrasound measurements of increment in muscle thickness. Statistically significant differences in pelvic floor muscle function measured by strength and thickness in the continent compared with incontinent nulliparous pregnant women.
**Nakamura et al.**^44^ **2014** Study design: Cross-sectional observational study Country: Brazil AIM: To determine how parturient women tolerate the use of a perineal distensibility assessment technique using the EPI-NO device.	Sample size: A total of 227 parturient. 110 were multiparous, and 117 were primiparous. Inclusion criteria: Full-term with a single, vertex fetus and exhibiting up to 9 cm of dilatation, at a maximum station of zero. Fetus showed good vitality. Primiparous and multiparous woman.	Interventions: A perineal distensibility assessment measured in cm with the inflated EPI-NO balloon. During the evaluation with EPI-NO, patients were asked about their sensation of discomfort.	Comparisons: Multiparous and primiparous	Outcomes: Perineal distensibility and degree of discomfort	Results: The balloon circumference attained by primiparous women was lower than that of multiparous women. The multiparous group reported significantly lower discomfort than the primiparous group. In addition, greater the perineal distensibility, the lower the discomfort caused by the test.
**Obloza** and Toozs-Hobson45 2018 Study design: Prospective cohort study Country: UK AIM: To identify properties of the functional female pelvic floor during pregnancy that could help to predict the mode of delivery.	Sample size: 133 women were included: 55 nulliparous, 52 primiparous (previous one vaginal birth), and 26 primiparous previous one LSCS and planned for VBAC. Inclusion criteria: Singleton pregnancy, general good health with no pre-existing conditions i.e. connective tissues disorders, urinary incontinence or other medical entities that might have hindered their obstetric care and influenced delivery mode.	Interventions: Pelvic ultrasound assessment: -third trimester (32 weeks) -three measurements of the elevator hiatus (levator hiatal area, its anterior-posterior and transverse diameters.	Comparisons: nulliparous, primiparous (previous one vaginal birth), primiparous previous one LSCS	Outcomes: Mode of delivery.	Results: Measurements less distensibility in VBAC group regardless whether a successful VBAC was achieved or delivered by an emergency LSCS. Less distensibility in women who had CS delivery with the least distensible muscles in VBAC group. Distensibility of puborectalis muscle in lavator’s hiatal AP diameter showed lesser distensibility in VBAC group that delivered vaginally versus the control group, however those women delivered by emergency LSCS, had AP diameter was even smaller.
**O’Boyle et al.**^46^ **2002** Study design: Analytic-correlational study Country: USA AIM: To determine whether pregnancy alone has any influence on objective measures of pelvic organ support compared with age- and race-matched control subjects.	Sample size: 21 pregnant and 21 non-pregnant Inclusion/exclusion criteria: The study group consisted of active duty women (between the ages of 18 and 29) seen for routine prenatal or gynecologic care in the obstetrics and gynecology clinic.	Interventions: Subjects underwent pelvic organ support evaluation by use of the pelvic organ prolapse quantification (POPQ) The Pearson χ2 statistic was used for statistical analysis, with a p value of 5% set for significance.	Comparisons: -pregnant women -non-pregnant women	Outcomes: POP Q	Results: In nulliparous women, pregnancy is associated with increased POP Q stage compared with nonpregnant control subjects.
**Palmezoni et al.**^29^ **2017** Study design: Analytic-correlational study Country: Brazil AIM: The objective was to evaluate the pelvic floor muscles (PFM) in primigravidae and compare them with those in nonpregnant nulliparous women.	Sample size: 141 women, four groups: non- pregnant nulliparous (C), primigravidae in first trimester (1T), in second trimester (2T), and in third trimester (3T) Inclusion criteria: Group C: non-pregnant and able to contract the PFM, for 1T, 2T, and 3T: being able to contract the PFM, physiological pregnancy, one fetus alive .	Interventions: The participants were examined by digital palpation for pelvic floor muscle contraction using the Modified Oxford Scale, by measuring maximal vaginal squeeze pressure with a vaginal perineometer, and by measuring PFM maximal strength using a vaginal dynamometer.	Comparisons: - non- pregnant nulliparous (C), - primigravidae in their first trimester (1T), - primigravidae in their second trimester (2T), - primigravidae in their third trimester (3T)	Outcomes: digital palpation, perineometry, and dynamometry	Results: Our data allow us to point out that PFM strength in primigravidae is lower compared with nonpregnant nulliparous women.
**Paschoal et al.**^47^ **2019** Study design: Cross-sectional observational study Country: Brazil AIM: To compare measurements of pelvic floor muscle extensibility in pregnant women obtained through the Epi-no^®^ and perineal elasticity meter (PEM) devices.	Sample size: 62 healthy pregnant women, GW 35 and 40 weeks. Inclusion criteria: Pregnant women >20 years old or older	Interventions: Perineal extensibility was measured using the Epi-no^®^ and PEM devices. The assessments were performed in random order: 29 pregnant women were first assessed with Epi-no^®^ and then the PEM, and 33 were first assessed with the PEM and then Epi-no^®^.	Comparisons: group 1 – patients first assessed with the Epi-no^®^, and then with the perineal elasticity meter; group 2- patients first assessed with the perineal elasticity meter, and then with the Epi-no^®^.	Outcomes: Perineal extensibility	Results: Linear correlation between the two measurements. In general, the higher the score obtained with Epi-no^®^, the higher the value obtained with the PEM. Epi-no^®^ degree of extensibility: C1- perineums with greater restriction – up to 17.49 cm; C2- perineums with moderate extensibility – from 17.5 cm to 20.8 cm; C3- perineums with good extensibility – 20.9 cm. PEM assessments:
**Rogers et al.**^30^ **2017** Study design: Cohort study Country: USA AIM: The objective of this analysis was to comprehensively describe pelvic floor changes during pregnancy in a low-risk, nulliparous population of women.	Sample size: 630 Nulliparous midwifery patients Inclusion criteria: Eligible women were greater than 18 years of age, nulliparous, had a singleton pregnancy, were able to complete questionnaires in English or Spanish, and did not have serious medical problems necessitating physician care. Enrollment until 36 completed weeks of gestation.	Interventions: Investigation of POPQ, UI, FI and perineal pain - early in the first or second trimesters	Comparisons: In the third trimester in order to characterize functional changes throughout pregnancy.	Outcomes: Symptom severity scales and quality of life measures for pelvic floor dysfunction.	Results: During pregnancy, women experience worsening UI, FI and perineal pain. UI symptoms are associated with a negative impact on quality of life. Sexual activity decreases and POPQ stage does not change.
**Sacomori et al.**^31^ **2010** Study design: Analytic-correlational study Country: Brazil AIM: To examine the relationship between PFM strength and body self- perception in pregnant women; and, more specifically, to determine the influence of the number of pregnancies (primigravida vs multigravida) on the strength of contraction of the PFM and on body self-perception.	Sample size: 35 pregnant_women (18 primigravidas, 17 multigravidas)	Interventions: semi-structured interview for socio-economic, gynecological and obstetric information; semi-structured Questionnaire of Human Corporeality; Evaluation of PFMS with Oxford scale	Comparisons: _	Outcomes: Pelvic floor strength measured through manual palpation, and body self-perception using the Questionnaire of Corporeality and Human Sexuality.	Results: PFMS was positively correlated with schooling [rho (ρ) = 0.496] and body self-perception variables: Primigravidas found their bodies more beautiful and were more satisfied with their bodies. On a scale of 0 to 6, multigravid participants expressed a greater wish than primigravid participants to be thinner (p=0.03). It was found that the primigravidas had a higher grade of contraction than the multigravidae, although this difference was not significan**t.**
**Sigurdardottir et al.**^32^ **2011** Study design: Observational descriptive study Country: Turkey AIM: They hypothesize that: 1) PFM strength and endurance is significantly reduced by first delivery in general, and 2) changes in PFM strength and endurance are influenced by mode of delivery.	Sample size: 33 primigravid women with a singleton pregnancy. Inclusion criteria: a singleton pregnancy was confirmed and without any major anomalies being found. Aged ≥18 years, healthy and being able to understand Icelandic or English.	Interventions: Pelvic organ support changes were documented by using POP-Q system. PFMS examination, by MOS, and symptom assessment by Pelvic Floor Distress Inventory-Short Form (PFDI-20) were performed at three time points: first (T1), second (T2), and third trimester (T3).	Comparisons: First trimester (11–13 weeks) (T1), second trimester (24–26 weeks) (T2), and third trimester (35–37 weeks) (T3).	Outcomes: To reveal the changes in pelvic organ support by performing serial measurements via Pelvic Organ Prolapse Quantification (POPQ) system and to assess associated pelvic floor symptoms via validated questionnaires.	Results: A significant reduction in PFM strength (p<0.0001) and endurance (p<0.0001) was found for all participants after first childbirth. The reduction in strength was 20.1 hPa, 31.4 hPa, and 5.2 hPa in the normal vaginal, instrumental vaginal and acute cesarean groups, respectively. The difference was significant between normal vaginal and acute cesarean birth (p=0.028) and instrumental vaginal and acute cesarean birth (p=0.003).
**Stær-Jensen et al.**^48^ **2013** Study design: Prospective cohort study Country: Norway AIM: To investigate whether pregnancy affects levator hiatus dimensions and the position and mobility of the bladder neck and the levator ani muscle in nulliparous pregnant women.	Sample size: 274 nulliparous pregnant women at 21 weeks and 37 weeks of gestation Inclusion criteria: Women with a previous pregnancy of more than 16 weeks duration, ongoing multiple pregnancy, or serious illness were excluded. Scandinavian-speaking women older than age 18 years were included.	Interventions: levator hiatus dimensions and the position and mobility of the bladder neck and the levator ani muscle were examined, using three-dimensional and four-dimensional transperineal ultrasonography at rest, during contraction, and during Valsalva maneuver.	Comparisons: Changes during pregnancy at 21 and 37 weeks of gestation	Outcomes: levator hiatus dimensions and the position and mobility of the bladder neck and the levator ani muscle	Results: An increase of all hiatal dimensions as well as bladder neck mobility was found from 21 weeks to 37 weeks of gestation in nulliparous pregnant women.
**Tennfjord et al.**^33^ **2014** Study design: Prospective cohort Country: Norway AIM: To compare vaginal resting pressure (VRP), PFM strength and endurance in women with and without dyspareunia, and to assess the impact of confounding variables on dyspareunia and PFM function.	Sample size: 177 nulliparous women Inclusion criteria: being a healthy, nulliparous, and singleton pregnant woman, speak Scandinavian language.	Interventions: Assessments of VRP, PFM strength, and endurance were performed at gestational week 22, and at 6 and 12 months postpartum. During the first clinical visit in women with dyspareunia	Comparisons: Women without dyspareunia	Outcomes: PFM. VRP, PFM strength, and endurance were assessed;	Results Twenty-eight and 30 % of the women reported dyspareunia at pre-pregnancy and at gestational week 22, respectively. At gestational week 37, and 6 and 12 months postpartum, the percentages were 40, 45, and 33 respectively. No difference in PFM variables was found between women with and those without dyspareunia.
**Toozs-Hobson et al.**^49^ **2008** Study design: Prospective observational cohort Country: UK AIM: To investigate and compare the effects of different modes of delivery on urethral sphincter volume, bladder neck mobility, and changes to levator hiatus distensibility using ultrasound imaging.	Sample size: 600 women were approached about this study. 110 of these attended for postpartum follow-up. Inclusion criteria: Primigravid women between 32 weeks and term.	Interventions: ultrasound pelvic floor assessment: -between 32 weeks and term -6 weeks postpartum -6 months postpartum.	Comparisons: -Vaginal delivery -Cesarean section	Outcomes: ultrasound variables from antenatal ‘baseline’ values at 6 weeks and 6 months postpartum	Results: The urethral sphincter was smaller after delivery compared to the third trimester. Vaginal delivery was associated with a significantly larger levator hiatus area on valsalva antenatally and at rest, squeeze, and valsalva postnatally compared to cesarean section.
**van Veelen et al.**^50^ **2014** Study design: Observational descriptive study Country: Netherlands AIM: To describe changes in the absolute values of levator_hiatal dimensions at rest, on pelvic floor contraction and on Valsalva maneuver using 3D/4D transperineal ultrasound in women during and after their first pregnancy.	Sample size: 231 nulliparous women with a singleton pregnancy	Interventions: All participants were invited for 3D/4D transperineal ultrasound examination at 12 and 36 weeks’ gestation. Volume imaging datasets were obtained at rest, on maximum pelvic floor muscle contraction and on maximum Valsalva maneuver.	Comparisons: 6 months postpartum	Outcomes: Variation in distensibility during pregnancy	Results: At 36 w, the absolute values of hiatal dimensions and the contractility and distensibility of the levator hiatus were significantly increased compared_with those at 12 w. Women who delivered vaginally showed a persistent significant increase in hiatal dimensions on Valsalva.
**Wijma et al.**^34^ **2001** Study design: Analytic-correlational study, prospective longitudinal study Country: Netherlands AIM: To assess the prevalence and the development of urinary incontinence in nulliparous pregnant women, and to investigate the relation of incontinence with the mobility of the urethro-vesical junction.	Sample size: 144 woman Inclusion criteria: -women ranged in age from 17 to 41 years -nulliparous - no history of incontinence, pelvic operations or neurological disease. -written informed consent	Interventions: Urinary incontinence was measured by a questionnaire and by a 24-hour pad test. The position of the urethrovesical junction and its mobility were measured by perineal ultrasound.	Comparisons: - 117 nulliparous pregnant women - 27 nulliparous non-pregnant controls	Outcomes: -Prevalence of urinary incontinence -Mobility of the urethro-vesical junction, indicated by the displacement/pressure coefficient	Results: Perineal ultrasound of the urethro-vesical junction showed lowering of the pelvic floor occurring as early as 12-16 weeks of pregnancy. Serial measurements of the displacement/pressure coefficient suggest that the dynamic characteristics of the connective tissues of the pelvic floor remain unaltered, whereas a significant decrease in pelvic floor muscle contraction occurs.
**Wu et al.**^11^ **2021** Study design: Prospective cohort study Country: USA AIM: Women during their first term pregnancy to elucidate the nature and timing of changes to the pelvic floor during pregnancy and after vaginal delivery.	Sample size: A total of 12 subjects were included in the final analysis. Inclusion criteria: ultrasound-confirmed singleton first trimester (within 14 wg) pregnancy, maternal age 18 and 40 years, English-speaking, and plans to deliver at Scott and White Medical Center. Vaginal delivery.	Interventions: Evaluation during the first trimester and late third trimester (at 37 weeks gestation). Assessment involved dynamic MRI imaging, a pelvic examination to measure prolapse using the Pelvic Organ Prolapse Quantification (POP-Q) scale.	Comparisons: Evaluation within a week of delivery, and 3 months postpartum	Outcomes: - dynamic MRI imaging, - pelvic examination to measure prolapse using the Pelvic Organ Prolapse Quantification (POP-Q) scale, - validated symptom questionnaires	Results: Anatomic changes measured by dynamic MRI and POP-Q examinations demonstrate significant descent at 3 months postpartum. However, these anatomic changes did not significantly correlate with changes in symptoms.
**Zanetti et al.**^51^ **2016** Study design: Prospective observational single cohort study Country: Brazil AIM: To determine a cutoff value for pelvic floor distensibility measured using the Epi-no balloon, which could be used as a predictive factor for perineal integrity in vaginal delivery.	Sample size: 227 parturients, 117 nulliparous and 110 multiparous. Inclusion criteria: Single births in the cephalic presentation with up to 9.0 centimeters of dilatation and at a station of zero, who received anesthesia and whose fetus showed good vitality.	Interventions: Pelvic floor (comprising pelvic floor and perineum) distensibility assessment with Epi-no device.	Comparisons: Multiparous and primiparous	Outcomes: Perineal trauma Intact perineum	Results: Circumferences greater than 20.8 cm achieved using the Epi-no balloon are a predictive factor for perineal integrity in parturients.
**Zhao et al.**^35^ **2017** Study design: Analytic-correlational study Country: China AIM: To investigate the effect of water immersion delivery on increasing strength of pelvic floor muscle (PFM) and relieving pelvic floor disorders (PFDs) during postpartum period.	Sample size: 2749 vaginal-delivery primiparas in postpartum 6-8 weeks Inclusion criteria: Chinese women, aged 20 to 40 years, primipara, singleton pregnancy, delivery after more than 37 w, vaginal delivery, clean lochia, no serious complications	Interventions: In this study, there were 600 patients in water immersion group and 2149 patients in the Conventional Group. Questionnaire survey of SUI Determination of PFM strength: Oxford scale diagnosis of POP Kegel exercises.	Comparisons: Non water immersion With and without episiotomy	Outcomes: PFM strength scales of women at postpartum 6 to 8 weeks between the 2 groups	Results: The rate of episiotomy in water immersion delivery group was lower than that in conventional delivery group (p<0.01). The primiparas without having an episiotomy have higher PFM strength than those having an episiotomy for both groups (p<0.01). There was a negative correlation between the scale of PFM strength and SUI or POP, wherein the r-values were 0.135 and 0.435, respectively (p<0.01).

**Table 2:** Distensibility, strength, and tone features reported by studies involved in the scoping review on investigating pelvic floor muscle strength and tone in women during the childbirth pathway from March to October 2022 (N=40)

*Studies*	*Distensibility*				*Strength*				*Tone*			
	*Pregnancy*	*Labor and birth*	*Postnatal period*	*Normative data*	*Pregnancy*	*Labor and birth*	*Postnatal period*	*Normative data*	*Pregnancy*	*Labor and birth*	*Postnatal period*	*Normative data*
Ali et al.^38^ 2022	✓											
Botelho et al.^15^ 2010						✓	✓					
Brunelli et al.^39^ 2020		✓										
Caroci Ade et al.^16^ 2010					✓		✓					
Caroci Ade et al.^17^ 2014					✓		✓					
Çetindağ et al.^1^ 2021	✓				✓							
Chen et al.^40^ 2013	✓											
Driusso et al.^18^ 2020						✓						
Dua et al.^41^ 2009		✓		✓								
Elenskaia et al.^19^ 2011					✓		✓					
Falkert et al.^42^ 2013		✓										
Frederice et al.^20^ 2013					✓				✓			
Gachon et al.^52^ 2017	✓											
Gray et al.^21^ 2020						✓						
Guntiñas et al.^43^ 2022		✓										
Halle et al.^22^ 2020						✓						
Hilde et al.^23^ 2013						✓	✓					
Jean Dit Gautier et al.^36^ 2018	✓											
Karahan et al.^37^ 2018										✓		
Klein et al.^24^ 1997					✓							
Hock et al.^25^ 2021					✓							
Meyer et al.^26^ 2000							✓					
Meyer et al.^27^ 2017						✓						
Mørkved et al.^28^ 2004								✓	✓			
Nakamura et al.^44^ 2014		✓										
Obloza et al.^45^ 2018		✓										
O’Boyle et al.^46^ 2002	✓											
Palmezoni et al.^29^ 2017					✓							
Paschoal et al.^47^ 2019				✓								
Rogers et al.^30^ 2017					✓							
Sacomori et al.^31^ 2010					✓							
Sigurdardottir et al.^32^ 2011							✓					
Stær-Jensen et al.^48^ 2013	✓											
Tennfjord et al.^33^ 2014						✓						
Toozs-Hobson et al.^49^ 2008		✓										
van Veelen et al.^50^ 2014	✓	✓										
Wijma et al.^34^ 2001	✓				✓							
Wu et al.^11^ 2021	✓		✓									
Zanetti et al.^51^ 2016		✓										
Zhao et al.^35^ 2017							✓					

### Perineal strength

This review included 22 articles that investigated pelvic floor strength^1,15-35^, 14 analytic correlational studies^1,15-17,22,23,26,27,29-31,33-35^, 2 observational descriptive studies^25,32^, 1 case study^36^, 1 RCT^28^, and 1 systematic review^18^. Included studies originated from 12 countries: Brazil (n=5), Norway (n=5), Switzerland (n=2), Turkey (n=2), UK (n=1), Pakistan (n=1), China (n=1), France (n=1), USA (n=1), Hungary (n=1), Canada (n=1), and Netherlands (n=1). Sample sizes of included studies were less than 100 (n=5) and more or equal to 100 (n=15) participants.

*Description of perineal strength*

There are different definitions of perineal force in literature, but only three studies describe it. In Palmezoni et al.^29^, PFM strength is defined as the ability to perform a correct contraction, meaning a squeeze around the pelvic openings and the inward movement (lift) of the pelvic floor. PFM strength is defined as the maximum voluntary contraction, which happens when a person attempts to recruit as many fibers in a muscle as possible. In Hilde et al.^23^, PFM strength is defined as contraction without any movement of the pelvis or visible contraction of the gluteal, hip, or abdominal muscles; this article also defines PFM endurance as a sustained maximal contraction, quantified during the first 10 seconds. In Sigurdardottir et al.^32^, PFM strength is the ability to perform a correct PFM contraction as a squeeze around the urethra, vagina and rectum and an inward (cranial) and forward (ventral) lift of the muscle plate. The other 20 articles analyzed did not describe perineal strength but reported a description of other indicators indirectly associated. Sacomori et al.^31^ did not give a definition of perineal strength but showed that there was a significant correlation between the variable strength of the PFM contraction and some variables of body self-perception.

*Normative data*

Only two studies reported normal features data on PFMs. Mørkved et al.^28^ measured PFM strength in nulliparous women at 18–20 weeks of pregnancy. The mean pelvic floor muscle strength measured as vaginal squeeze pressure among continence women was 39.5 cm, whereas women with incontinence had mean vaginal squeeze pressure of 32.0 cm.

In Fredrice et al.^20^, the third degree on the Oxford scale was the maximal muscle strength exhibited by pregnant women (aged 18–35 between 30–34 weeks of pregnancy, one fetus).

*Assessment of perineal strength*

Perineal strength was measured in several ways, including vaginal digital examination to the modified Oxford Scale^1,15,16,17,19,26,29,31,35^; perineometry^16,17,19,24,25,29^; manometer/dynamometer^23,29,33^; translabial 2D/3D or transperineal ultrasonography^22,26,34^; POP-Q test^1^; intravaginal air-inflated balloon catheter^22,25,28^; and the device Myomed 932^® 32^ and Brink’s scale^30^ (Table 1).

*During pregnancy*

Eight articles analyzed the effects of pregnancy on pelvic floor muscle strength.

Hock et al.^25^ and Palmenzoni et al.^29^ are in agreement with the result regarding a decrease in PFM in pregnant women in correlation compared to non-pregnant women. Other studies that analyzed only variations of PFM during pregnancy did not find significant differences in strength among the stages of pregnancy^1,7^. Rogers et al.^30^ showed, with the Brinks scores, that the pelvic organ support did not change over the course of pregnancy. Wijma et al.^34^ and Elenskaia et al.^19^ confirm this thesis, showing that the dynamic characteristics of the connective tissues of the pelvic floor remain unaltered, although a significant decrease in pelvic floor muscle contraction occurs. Elenskaia et al.^19^ demonstrated a significant improvement in PFM function during pregnancy in this study, even if some women practice pelvic floor exercises during pregnancy. Klein et al.^24^ noticed a slight trend among women with perineal tears for having weaker pelvic floor strength in the antepartum period. Two studies compared PFMs women in their first pregnancy and previous birth, and all are in agreement on the reduction of PFMs in the second situation^17,31^.

*During labor and birth*

Nine articles analyzed the effects of labor and childbirth on pelvic floor muscle strength.

Many of the studies reviewed in this article agree that pelvic floor strength changes significantly after birth. Multivariate analysis showed that birth mode was the most important factor for change in PFM variables^15,18,23,27,32,33^.

Literature shows that vaginal birth is more harmful to PFMs than cesarean section^15,23,32^, but there are contrasting opinions regarding the eutocic vaginal birth compared to the dystocic one: Mayer et al.^26^ reports that dystocic vaginal birth is more harmful for PFMs than spontaneous vaginal birth. Sigurdattodir et al.^32^ and Caroci et al.^16^ report that PFMs were not significantly influenced by mode of birth (instrumental birth or severe perineal trauma).

In addition, Zhao et al.^35^ found that water birth is associated with a lower episiotomy rate, and avoiding episiotomy is beneficial for maintaining the PFM strength of women in postpartum.

Three studies are more focused on the anatomical and functional aspects of PFMs.

Hilde et al.^23^ report that in addition to a general weakness of the PFM, muscle, peripheral nerve, and connective tissue injuries may play an important role in the reduction of PFM function.

Halle et al.^22^ suggest that antenatal PFM training might improve the ability of levator ani muscle (LAM) to facilitate birth and resist major LAM defects.

The study of Gray et al.^21^ provides a better understanding of the roles of the muscles involved in the second and third stages of labor, reaching an important conclusion for scientific literature about PFM: contractility of PFMs contributes to normal labor processes and deserves further investigation.

*Postnatal period*

Six articles analyzed the effects of postpartum on pelvic floor muscle strength.

Most PFM strength studies agree that childbirth leaves consequences in the postnatal period^15,16,26,32,35^, such as low intensity in PFM strength^15,16,^^32^ and endurance32 or decrease in intra-anal pressure in forceps delivery^26^. Episiotomy reduces PFM strength^35^.

In particular, Hilde et al.^23^ say that comparing women with spontaneous vaginal birth and instrumental vaginal birth, there were no significant differences in changes from mid-pregnancy to 6 weeks postpartum in PFM strength. One study found that avoiding episiotomy is beneficial for maintaining the PFM strength of women in postpartum 6–8 weeks, and the strength of PFMs during the postpartum period can be improved by doing the Kegel exercise^35^.

### Perineal tone

We found three articles that studied the pelvic floor tone^20,28,37^: one RCT^28^ and two Analytic-correlational studies^20,37^. Included studies originating from 3 countries: Brazil, Turkey, and Norway. Sample sizes of included studies ranged from 1 to 100 (n=1) and 100–500 (n=2) participants.

*Description of pelvic floor muscle tone*

None of the studies reviewed gives a definition of pelvic floor muscle tone.

Two of those^20,37^ analyzed PFM contractile components even though there is no agreement on naming. Karahan et al.^37^ call it ‘tonic activity’, and Fredrice et al.^20^ call it ‘basal tone’ (BT), while they call tonic fibers the average sustained contraction (ASC). Both studies^20,37^ measure the resting electrical activity of PFMs by PFM surface electromyography (sEMG).

Another study^28^ indirectly investigates pelvic floor muscle tone by analyzing the viscoelastic component through measurements of the thickness of the urogenital diaphragm using perineal ultrasound.

*Normative data*

Karahan et al.^37^ point out that PFMs are the only skeletal muscles that exhibit continuous low-level myoelectric activity in a resting state commonly referred to as ‘tonic’. This tonic muscular activity continues without conscious awareness, but it can also be inhibited voluntarily. It is very important that the PFMs also exhibit stronger voluntary or reflex contractions of short duration, called ‘phasic’, in response to pain or sudden increases in intra-abdominal pressure. Pain and increased intra-abdominal pressure are typical components of labor. Therefore, higher level ‘phasic’ PFM activities might occur during labor. On the other hand, inhibition of the tonic activity of the PFMs, which leads to muscle relaxation to allow evacuation of the contents of the pelvic organs, should also be expected to occur during labor, especially during uterine contractions (UC), just as during bladder or rectal contractions at the time of expulsion or defecation.

Mørkved et al.^28^ underline that the muscular layers form structural support, the pelvic floor muscle volume influences the anatomical location of the pelvic organs, and a fast and strong contraction of the pelvic floor muscles ensures continence during an abrupt increase in abdominal pressure.

*During pregnancy*

One study^20^ demonstrated a high prevalence of urinary symptoms in the late third trimester of nulliparous women, but no association was found between urinary symptoms and maximum voluntary contraction (MVC), average sustained contraction (ASC), and PFM strength, except basal tonus. They demonstrated that irritative bladder (IB) symptoms were associated with a decreased BT. Despite this, they found that some individuals with low resting tension, when requested to contract voluntarily, prove capable of contracting very efficiently.

Another study^28^ measured pelvic floor muscle function by assessing muscle strength and thickness in continent and incontinent nulliparous pregnant women. In addition, they found a possible correlation between measurements of pelvic floor muscle strength and thickness in nulliparous pregnant women at 20 weeks of pregnancy. The superficial pelvic floor muscles of continent women were statistically significantly thicker compared to the incontinent women both during relaxation and contraction.

*During labor and birth*

Karahan et al.^37^ investigated the behavior of PFMs during uterine contractions (UC) in spontaneous and oxytocin-induced labor in term pregnant women. The data show a prevalence of higher-level ‘phasic’ activity of PFMs during UC in the group of women who received oxytocin during labor, while PFMs remained relatively silent in women with spontaneous birth.

It can be hypothesized that this relatively silent state of PFMs is the predominant behavior of PFMs during uninduced contractions.

Since PFMs exhibit contractions in response to pain, more intense and more painful contractions in the oxytocin group may explain the higher rate of PFM contractions.

In addition, multiparous women showed more PFM contractions per UC than nulliparous women, both in oxytocin-treated and spontaneous labor groups.

*Postnatal period*

No studies were found regarding pelvic floor muscle tone during the postpartum period.

### Perineal elasticity

This review included nineteen articles that investigated pelvic floor elasticity and/or distensibility^1,8,11,34,36,38,49-51^: seven prospective observational cohort studies^11,39,40,45,48,49,51^, two cross-sectional observational studies^44,47^, one prospective observational study^41^, one prospective longitudinal study^8^, two observational descriptive studies^42,50^, five analytic-correlational studies^11,34,38,43,46^ and one case study^36^.

Included studies originated from twelve countries: UK (n=3), Brazil (n=3), USA (n=2), France (n=2), Netherlands (n=2), Spain (n=1), Norway (n=1), Germany (n=1), Italy (n=1), China (n=1), Turkey (n=1) and Pakistan (n=1). Sample sizes of included studies ranged from 1 to 100 (n=8), 100–500 (n=9), and 500–1000 (n=2) participants.

*Description of distensibility*

Seven studies defined the distensibility of the pelvic floor muscles using different terms.

Distensibility of the levator ani muscle was defined as the ratio between the area of the hiatus on Valsalva and the area of the hiatus at rest^8,43,45,50^. Additionally, van Veelen et al.^52^ measured the levator hiatal transverse diameter and the area of the levator hiatus. Zanetti et al.^53^ assessed the pelvic floor distensibility (comprising pelvic floor and perineum) as the circumference in centimeters of the inflated balloon of the Epi-no device. Two authors considered the extensibility of pelvic floor muscle defined as the intensity of the sensation of discomfort perceived by women through Epino^44,47^ and PEM devices^47^.

Twelve studies did not describe elasticity but reported a description of other indicators indirectly associated with elasticity^1,11,34,35,38,39-42,46,48,49^.

The pelvic floor distensibility was measured in several ways, including transperineal ultrasound^15,34,38,39,42,45,49,50,52^, magnetic resonance imaging^36^, POP-Q system^1,40,46^, tape42 EPI-NO balloon^11,44,47,51^ and PEM device^47^.

### Normative data

One study provided normative data on perineal length (Caucasian and Asian women). The mean perineal length in Caucasian women was 3.7 cm, and in Asian women, it was 3.6 cm, assessed during the first stage of labor. In conclusion, they have established a strong correlation between short perineal length and third-degree tears^41^.

Paschoal et al.^47^ classified perineal distensibility into three categorical groups according to their degree of extensibility measured with Epi-no^®^. In relation to the perimeter measurements obtained from the Epi-no^®^ balloon, the mean was 18.9 cm.

One study evaluated perineal distensibility tolerance during labor using EPI-NO devices. The tolerance of its use was directly correlated to the patient’s perineal distensibility, which is greater in multiparous than in primiparous patients^44^.

*During pregnancy*

Ten articles^1,11,34,35,38,40,46,48,50,52^ analyzed the effects of pregnancy on pelvic floor distensibility. Stær-Jensen et al.^48^ found an increase in all hiatal dimensions as well as bladder neck mobility from 21 weeks to 37 weeks of gestation in nulliparous pregnant women.

Similarly, van Veelen et al.^50^ showed an increase in the absolute values of hiatal dimensions and in contractility and distensibility of the hiatus between 12 and 36 weeks’ gestation.

Çetindağ et al.^1^ observed during pregnancy a significant descent both in all compartments of the vaginal wall and perineum with an increase in total vaginal length throughout the first pregnancy of women. Similarly, Gachon et al.^52^ found an association between peripheral ligamentous laxity and pelvic organ mobility.

A weakness of the connective tissue, measured through the POP-Q system, was observed in the third trimester of pregnancy^38^. In addition, Chen et al.^40^ suggested that significant stage II pelvic organ prolapse (POP) occurs during the third trimester of pregnancy in low-risk nulliparous women. Similarly, a case-control study^46^ showed that the POP-Q stages were significantly higher among pregnant women, indicating more evidence of pelvic prolapse.

Wu et al.^11^ supported the significant pelvic floor changes (bladder neck descent, increased genital hiatus, and POP-Q stage) that occur after birth when measured three months postpartum compared to during pregnancy at multiple time points and the immediate postpartum period.

Perineal ultrasound of the urethrovesical junction showed lowering of the pelvic floor occurring as early as 12–16 weeks of pregnancy^34^. There is an increase in the length of uterosacral ligaments at 16, 32, and 38 weeks^35^.

*During labor and birth*

Some studies described how pelvic floor distensibility affects intrapartum outcomes. One study found a significant negative relationship between the change of the anteroposterior diameter of levator hiatus from rest to Valsalva in nulliparous women at term and the duration of the active second stage of labor: the higher the increase of APD with Valsalva, the shorter was the active second stage of labor. This association was independent, even when adjusting for the use of epidural analgesia^39^.

One study evaluated pelvic floor distensibility during the first stage of labor and found a negative correlation between perineal length and third-degree tear in primigravid women. Authors concluded that there is a higher incidence of third-degree perineal tears in women with short perineum^41^. Short perineal length is strongly correlated with third-degree tears^41^. Higher distensibility with Epi-no balloon values larger than 20.8 cm was a predictive factor for perineal integrity^51^.

Some studies described how pelvic floor distensibility affected the mode of birth.

Obloza et al.^45^ described a higher distensibility in women who had vaginal birth compared to women with an emergency CS regardless of their previous mode of birth. Women who underwent a successful vaginal birth after caesarean section (VBAC) had less distensible pelvic floor muscles compared to their nulliparous and primiparous counterparts (who had vaginal birth in the past).

Vaginal birth is strongly associated with a larger, more distensible levator hiatus and a greater degree of bladder-neck mobility both antenatally and postpartum^49^. Women who had vaginal birth showed a persistent increase in hiatal dimensions on the Valsalva maneuver, whereas women who received a pre-labor or first-stage CS showed no significant changes in hiatal dimensions on the Valsalva maneuver. However, both women who had a spontaneous or CS showed a persistent increase in the distensibility of the elevator hiatus during the Valsalva maneuver compared with early pregnancy^50^.

The smaller hiatus area during pregnancy at rest and during Valsalva is associated with a higher risk of CS due to failure of labor progression. Women with vaginal birth had significantly larger hiatal areas compared with women delivered by cesarean section^42,43^.

*Postnatal period*

Only one study analyzed the distensibility in the postpartum period. The authors evaluated the bladder descent and elevator hiatus area at different time points during the gestation and postpartum period, during the Valsalva maneuver, and at rest. In the early postpartum period (a week after birth), they observed a bladder descent during the Valsalva maneuver and no statistically significant difference in the elevator hiatus area^11^.

## DISCUSSION

This scoping review succeeded in exploring the literature with the aim of clarifying what was known about changes in pelvic floor function in terms of strength and tone during the childbirth pathway (pregnancy, intrapartum, and postnatal period).

PFM strength has been extensively investigated in the current research landscape. Given the complex nature of the topic, a multiplicity of assessment tools has been used to evaluate it. The results of this scoping review highlighted a decrease in PFM strength in pregnant women compared to non-pregnant women, as well as a decrease in PFM strength in multiparous compared to primiparous women. Despite this, no significant differences in PFM strength were found across pregnancy states. In addition, most studies agree that childbirth leaves consequences in the postnatal period. Especially there is almost a total consensus that vaginal birth is more harmful to PFMs than cesarean section. Limited evidence was found regarding other modes of birth (instrumental vaginal birth). Moreover, few studies highlight the positive aspects of PFMs, such as avoiding episiotomy and doing exercises in the antenatal period. Having a deep understanding of intrapartum practices that may have a negative effect on PFM changes in short-, medium-, and long-term is a strategic element that should be taken into consideration in order to enhance maternal and neonatal outcomes. For this reason, further research is needed to explore the relationship between midwifery intrapartum care and pelvic floor muscle function in depth.

In contrast, there is a paucity of data in the literature regarding tone parameters, and even those that mention it do not give a definition. However, our results identified a correlation between decreased basal tone in pregnancy and urinary dysfunction in the late third trimester of pregnancy in nulliparous women. In addition, the superficial pelvic floor muscles of continence women were found to be statistically significantly thicker compared to the incontinent women both during relaxation and contraction. There is no agreement of findings regarding a possible correlation between strength and tone in PFMs. No articles investigated tone in the postpartum period. Only one study^19^ investigated PFM tone in relation to labor and birth. Further prospective studies with appropriate follow-up intervals are needed to assess the electrophysiological behavior of PFMs during labor contractions and associated clinical consequences. The physiological processes and changes in PFMs occurring during pregnancy should be considered by midwives in order to ensure antenatal and intrapartum care, promoting a normal adaptation and preventing damage related to inappropriate intrapartum interventions such as episiotomy. Additionally, to ensure a high quality of maternity care during the postpartum period, an in-depth analysis of the association between prompt screening and pelvic dysfunction in the medium- and long-term is mandatory.

Moreover, our findings identified another fundamental key concept besides those of PFM strength and tone: distensibility. A large number of studies investigated this aspect of pelvic floor function, and a large number of indicators were considered. Although there is no consensus of definition and consequently neither of assessment method, our results report concordance in the following points: there is an increase of PFM distensibility during pregnancy; there is an association between peripheral ligamentous laxity and pelvic organ mobility; and higher POP-Q stages were found among pregnant women. In addition, pelvic floor distensibility is greater in multiparous than in primiparous women. Notably, it was shown that pelvic floor distensibility affects intrapartum outcomes as well as the mode of birth.

Only one study^37^ analyzed the distensibility in the postpartum period, underling a bladder descent during the Valsalva maneuver in the early postpartum period (a week after vaginal birth). A single study^9^ reported data on normality.

### Strengths and limitations

The strengths of this review include the co-participation of a multidisciplinary group of experts in the osteopathic, physical therapy, midwifery, and obstetrical areas. The multidisciplinary approach improved the quality of the research, minimizing bias and increasing the validity and reliability of the results. Each member of the group brought his or her own skills, knowledge, and experience to the research project. This diversity allowed for a more comprehensive and nuanced understanding of the research problem, which enabled elements not initially included to be considered. Thanks to this multidisciplinary team, the research findings can be disseminated to a wider audience and have a greater impact on patient care.

The review includes only articles published in English and Italian, and this could be a limitation of the review as other studies written in other languages may have been excluded. Moreover, the conclusions of the review were written months after the last articles were selected, so relevant studies may have been published in the timeframe not considered.

## CONCLUSIONS

This scoping review provided a mapping of the existing literature on the functionality of PFMs, identified key concepts, and provided an overview of the current state of research in this field. Compared with the initially considered parameters of strength and tone of PFMs, distensibility emerged as an equally important key concept; however, in all three areas (strength, tone, and distensibility), a lack of clarity on the most reliable assessment tools emerged.

Areas of consensus and disagreement were found in all three themes analyzed throughout the entire birth pathway (pregnancy, labor, mode of birth, and postpartum period).

In particular, this review revealed a paucity of data regarding the tone of PFMs during the entire pathway of childbirth, underscoring the need for further studies on this issue. Moreover, although the concepts of strength and distensibility have been addressed more in the literature, further studies investigating the labor and postpartum period are needed.

## Supplementary Material



## Data Availability

The data supporting this research are available from the authors on reasonable request.
