# Supplementary material for: What is known about changes in pelvic floor muscle strength and tone in women during the childbirth pathway? A scoping review
Source: Eur J Midwifery. 2024 Aug 2;8:10.18332/ejm/189955. doi: 10.18332/ejm/189955 (PMC11295251; doi:10.18332/ejm/189955)
Supplement: Supplementary file 1 [file EJM-8-42-s1.pdf]

Supplementary Table 1

PCC
POPULATION: healthy women in pregnancy or labor and birth or postnatal period
CONCEPT: changes in pelvic floor muscle (strength and tone) during the childbirth pathway.
CONTEXT: medium and high income countries. Hospital and community midwifery care.

Database	Search strategy	Records
Pubmed, OVID, Medline, ScienceDirect, The Cochrane Central Library, Scopus, Web of Science, PEDro, Scholar Google, Embase, CINHAIL.	*(((((perineum*[MeSH Terms]) OR (perineum stress[Title/Abstract])) OR (perineum muscle strength[Title/Abstract])) OR (perineum muscle tone[Title/Abstract])) OR (perineum disorder[Title/Abstract])) AND (birth*[MeSH Terms])*	1731

© 2024 Manzotti A. et al.
